# Rapid Method for the Determination of 5-Hydroxymethylfurfural and Levulinic Acid Using a Double-Wavelength UV Spectroscopy

**DOI:** 10.1155/2013/506329

**Published:** 2013-10-08

**Authors:** Junhua Zhang, Junke Li, Yanjun Tang, Guoxin Xue

**Affiliations:** ^1^Engineering Research Center for Eco-Dyeing and Finishing of Textiles, Ministry of Education, Zhejiang Sci-Tech University, Hangzhou 310018, China; ^2^Key Lab. of Biomass Energy and Material, Nanjing 210000, China

## Abstract

This study reports on a rapid method for the determination of levulinic acid (LA) and 5-hydroxymethylfurfural (HMF) in acid hydrolyze system of glucose based on UV spectroscopy. It was found that HMF and LA have a maximum absorption at the wavelengths of 284 nm and 266 nm, respectively, in a water medium, and the absorptions of HMF and LA at 284 nm and 266 nm follow Beer's law very well. However, it was found that a major spectral interference species will arise in the quantification of HMF and LA; nonetheless, this interference can be eliminated through the absorption treatment of charcoal. Therefore, both HMF and LA can be quantified with a double-wavelength technique. The repeatability of the method had a relative standard deviation of less than 4.47% for HMF and 2.25% for LA; the limit of quantification (LOQ) was 0.017 mmol/L for HMF and 4.68 mmol/L for LA, and the recovery ranged from 88% to 116% for HMF and from 94% to 105% for LA. The present method is simple, rapid, and accurate. It is suitable to use in the research of the preparation of HMF and LA in biorefinery area.

## 1. Introduction

Levulinic acid (LA) can be used as a new platform chemical for the production of a wide range of value-added products through salification, esterification, hydrogenation, condensation, oxidation and halogenation reaction [1, 2]. So, it will have important stratagem significance for the preparation of LA from biomass resources, especially from non-foodstuff resources [3]. The preparation of LA often requires the use of stalk or the solid abandon matter which contains cellulose as the origination material and was first hydrolyzed to glucose or fructose at high temperature, and the formed sugar was dehydrated to LA via a platform chemical of HMF [4, 5]. Therefore, understanding the production law of HMF and LA in the acid hydrolyzed system of glucose or fructose will have an important significance, and the concentration measurement of HMF and LA in the acid hydrolyzed system will be the emphasis premise.

The traditional quantitative analysis for HMF included thiobarbi acid method [6] and thiosemicarbazone method [7]. However, thiobarbi acid method should remove the deposit that was generated during the Winkler reaction; thiosemicarbazone method in the sample should be distilled, and both methods need chromogenic reaction before analysis [8]. In recent years, ion chromatography (IC), high performance liquid chromatography (HPLC) [9–12], and gas chromatography (GC) [13–16] also have been used for the analysis of HMF and LA; however, these instruments are expensive and the relative maintenance costs are high.

In this work, we have developed a UV spectroscopic method for the simultaneous determination of HMF and LA. The present method is simple, rapid, and accurate and has the potential for online process monitoring.

## 2. Experimental Section

### 2.1. Chemicals

All chemicals used in the experiments were from commercial sources. Five HMF solutions (the concentration range is from 0 to 0.1 mmol/L) and LA solutions (the concentration range is from 0 to 65 mmol/L), analytica1 grade, were used as the standard for calibration. A 5 wt% of H_2_SO_4_ solution was used to hydrolyze glucose.

### 2.2. Samples

Seven stream samples were collected from the H_2_SO_4_ solution hydrolyze system of glucose in the laboratory using a reaction kettle. The process conditions of acid hydrolyze system experiments were as follows: 3 g of glucose was used, 50 mL of H_2_SO_4_ solution (5 wt%) was poured into the reaction kettle, the reactor was placed in the electricity bath and heated to 180°C, and time was recorded from the set-value temperature. The reaction was stopped after 2 h from the start of the reaction and cooled to room temperature.

### 2.3. Apparatus

A UV-Vis spectrophotometer (S-3100, Shinco, Korea) equipped with a 1 cm path length flow cell was used for the experiments.

### 2.4. Procedures

Calibration was conducted by preparing a set of standard solutions, that is, 0.019, 0.037, 0.056, 0.075, and 0.093 mmol/L of HMF and 20.25, 29.80, 39.00, 47.86, and 64.66 mmol/L of LA. The absorption spectrum for each solution was measured at wavelength of 284 nm and 266 nm, respectively.

For a typical UV analysis of glucose hydrolysate, 5 mL of filtrate for glucose hydrolysate and 0.5 g of activated charcoal were added to a 10 mL of colorimetric tube. The solution was boiled for 1 min; then, the reaction solution was filtrated by filter paper, and the filtrate was measured at the wavelength of 284 nm and 266 nm after filtration.

## 3. Results and Discussion

### 3.1. Spectral Characteristics of HMF and LA Complex

**Figure 1:**
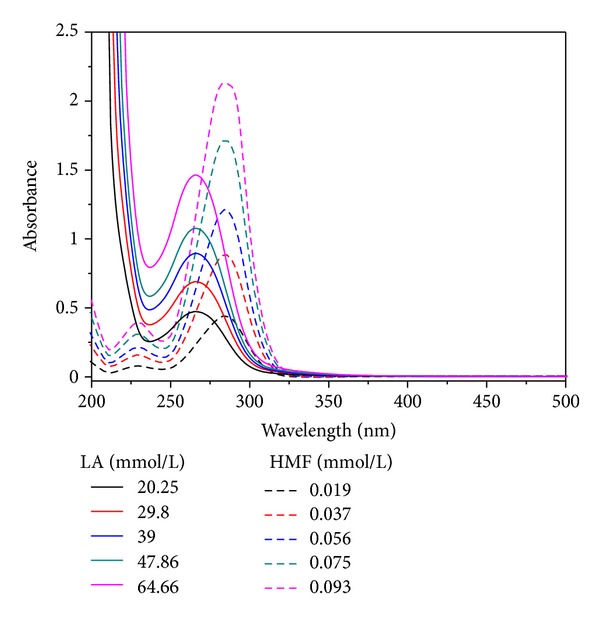
Spectra of HMF and LA.

UV light can be absorbed by HMF and LA. Therefore, HMF and LA can be determined by spectroscopy as long as there is no spectral interference. As shown in Figure 1, HMF and LA have strong absorption in the UV range below 330 nm, and their characteristic absorption is at wavelength of 284 nm and 266 nm, respectively. Thus, the concentration of HMF and LA can be measured.

**Figure 2:**
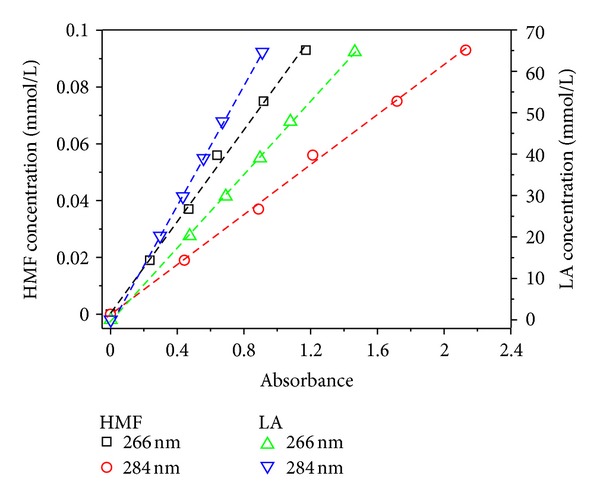
Calibration curves for HMF and LA.

As shown in Figure 2, the absorptions of HMF and LA at 266 nm and 284 nm follow Beer's law very well. The molar absorptivity at the wavelength of 266 nm and 284 nm is 12.38, 22.7 mmol^−1^·L·cm^−1^ for HMF and 0.023, 0.014 mmol^−1^·L·cm^−1^ for LA, respectively. And the standard calibration curve was obtained; that is,

(1) AHMF,266=−0.0055(±0.021)+12.38(±0.37)C(n=6,r2=0.9954),

(2) AHMF,284=0.006(±0.029)+22.7(±0.5)C(n=6,r2=0.9975),

(3) ALA,266=0.0096(±0.0077)+0.023(±0.0002)C(n=6,r2=0.9996),

(4) ALA,284=0.0075(±0.0058)+0.014(±0.0001)C(n=6,r2=0.9995),

where *A*_HMF,266_,  *A*_HMF,284_,  *A*_LA,266_,  *A*_LA,284_, and *C* represent, respectively, the UV signal response for HMF and LA at 266 nm and 284 nm and the HMF and LA concentration (in mmol/L) of the standard samples for HMF and LA. It can be seen from (1) to (4) that there is a good linear relationship at the linear range of 0−0.093 mmol/L for HMF and 0–64.66 mmol/L for LA.

Being calculated by (5) [17, 18], the limit of quantitation (LOQ) in the present method is 0.017 mmol/L for HMF (at 266 nm) and 4.68 mmol/L for LA (at 284 nm), which can meet the requirements for glucose hydrolysate test:

(5) $$\begin{array}{c} \text{LOQ}=\frac{a+10\times \left|\Delta a\right|}{s}, \end{array}$$

where *a*,  Δ*a*, and *s* respresent the intercept, uncertainty of the intercept, and the slope in (1) to (4), respectively.

### 3.2. Spectral Interference

**Figure 3:**
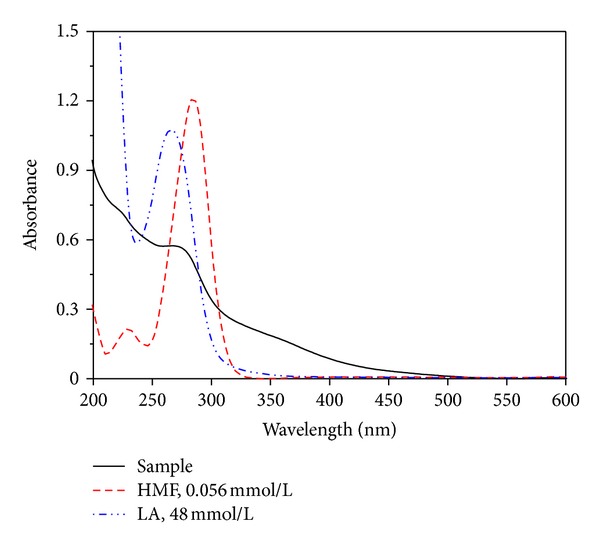
Spectra of HMF, LA, and sample.

For HMF and LA determination in hot acid hydrolysis solution of glucose or fructose, the major spectral interference species are produced by byproducts which are generated by subsidiary reactions during the hot acid hydrolysis. As shown in Figure 3, the standard samples of HMF and LA only have absorption in the UV range below 350 nm; however, the hot acid hydrolysis solution has an obvious absorption in the UV range at wavelengths between 350 nm and 450 nm. Therefore, the byproducts of the hot acid hydrolysis solution interfere in the determination of HMF and LA by UV spectrophotometry. Fortunately, the charcoal can be used to eliminate the interference. Therefore, the interference of byproducts in HMF and LA determination using the spectral characteristics at wavelengths of 266 nm and 284 nm can be neglected.

### 3.3. The Elimination of Spectral Interferences

#### 3.3.1. The Effect Determine of the Charcoal Absorption

**Figure 4:**
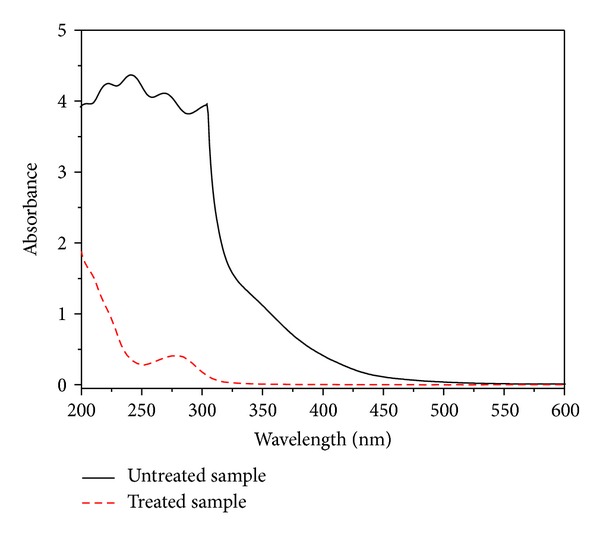
Spectra of the sample before and after it was treated by charcoal.

The spectral difference of a sample before and after it was treated by charcoal was shown in Figure 4; it can be seen that the absorption in the UV range at wavelengths between 350 nm and 450 nm had been eliminated after the adsorption treatment by charcoal. Therefore, the interference of byproducts can be eliminated, and the absorbance of HMF and LA can be determined.

#### 3.3.2. The Dosage of Charcoal Required for the Absorption

**Figure 5:**
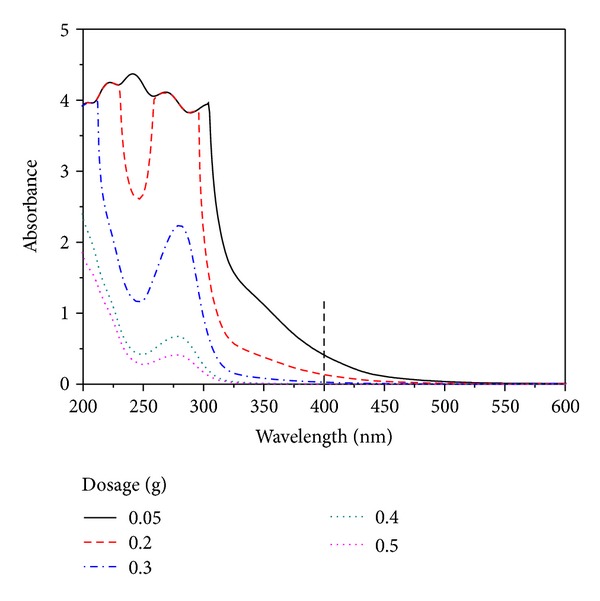
Effect of charcoal dosage on the spectra of a sample.

The dosage of charcoal can affect the adsorption ratio of byproducts. So, it will influence the spectra of the sample after adsorption treatment by charcoal. As shown in Figure 5, the absorbance of an acid hydrolysis sample at wavelengths between 200 nm and 600 nm gradually dropped along with the enhancement of charcoal dosage.

**Figure 6:**
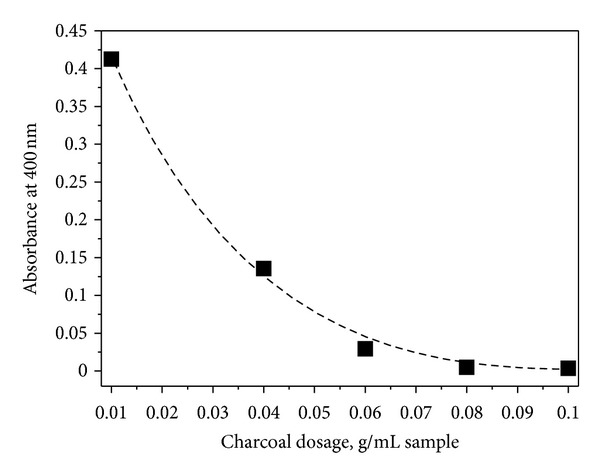
Effect of charcoal dosage on the absorbance at 400 nm of a sample.

The absorption intensity at wavelengths of 400 nm as a function of charcoal dosage, which indicated that complete interference was eliminated when the dosage of charcoal achieved 0.1 g/mL sample (Figure 6). If a further reduction in charcoal dosage is desired, increasing the boiling time can be an option since the adsorption ratio should increase in a long time. However, to avoid further hydrolysis of LA and HMF and to find the proper charcoal dosage at higher temperature, a curve similar to that in Figure 6 should be established. Overall, the present procedure is simpler and faster. It involves a single reaction step and requires less chemicals and analytical equipment.

#### 3.3.3. The Calibration Coefficient Determine of HMF and LA

**Figure 7:**
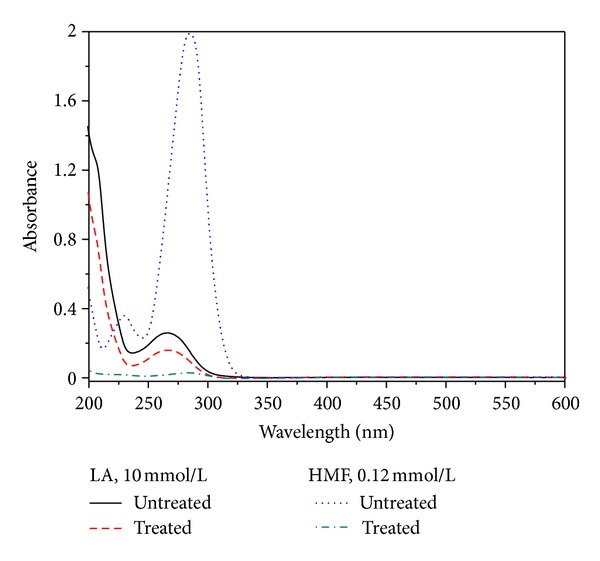
Effect of charcoal absorption on the absorbance of LA and HMF.

During the charcoal absorption treatment of the hot acid hydrolysis solution, not only the byproducts were absorbed, but also HMF and LA would be absorbed at a certain extent.  Figure 7 shows the spectrograms of HMF and LA standard solutions before and after they were treated by charcoal. So, the calibration coefficients of HMF and LA can be determined, which can be derived as

(6) $$\begin{array}{c} K_{\text{HMF}}=\frac{A_{b,284}}{A_{a,284}},\\ K_{\text{LA}}=\frac{A_{b,266}}{A_{a,266}}, \end{array}$$

where *K*_HMF_, *K*_LA_ are the calibration coefficients of HMF and LA at wavelengths of 284 nm and 266 nm, respectively. *A*_*b*,284_ and *A*_*a*,284_ are the absorbance of HMF at 284 nm before and after the standard solution was treated by charcoal. *A*_*b*,266_ and *A*_*a*,266_ are the absorbance of LA at wavelengths of 266 nm before and after it was treated by charcoal. The calibration coefficients *K*_HMF_ and *K*_LA_ are 69.3 and 1.62, respectively, which was obtained from the calibration graph shown in Figure 7.

### 3.4. A Dual-Wavelength Method to Determine the Content of HMF and LA

In this paper, we developed a dual-wavelength spectrophotometric method to determine the contents of HMF and LA at the same time. As Figure 1 had shown previously, HMF and LA in the hot acid hydrolysis solution had the characteristic absorption at wavelengths of 284 nm and 266 nm, respectively. Meanwhile, the absorbance at the scope of 250 nm and 350 nm was the contribution of HMF and LA. Based on Beer's law, the concentration of HMF and LA in the sample can be calculated according to the following equation:

(7) $$\begin{array}{c} A_{266}=\varepsilon_{\text{LA}}^{266}C_{\text{LA}}+\varepsilon_{\text{LA}}^{284}C_{\text{HMF}},\\ A_{284}=\varepsilon_{\text{HMF}}^{266}C_{\text{LA}}+\varepsilon_{\text{HMF}}^{284}C_{\text{HMF}}, \end{array}$$

where *A*_266_ and *A*_284_ are the absorbance after the sample was treated by charcoal at wavelengths of 266 nm and 284 nm, respectively. *C*_LA_  and *C*_HMF_ are the diluted concentrations of LA and HMF in the sample after the sample was treated by charcoal, mmol/L. And *ε*_LA_^266^, *ε*_HMF_^266^,  *ε*_LA_^284^, and *ε*_HMF_^284^ are the molar absorptivities of LA and HMF at wavelengths of 266 nm and 284 nm, respectively, which can be achieved from Figure 2. Then, the *C*_LA_ and *C*_HMF_ can be written as

(8) $$\begin{array}{c} C_{\text{LA}}=\frac{\varepsilon_{\text{HMF}}^{284}A_{266}-\varepsilon_{\text{LA}}^{284}A_{284}}{\varepsilon_{\text{LA}}^{266}\varepsilon_{\text{HMF}}^{284}-\varepsilon_{\text{LA}}^{284}\varepsilon_{\text{HMF}}^{266}},\\ C_{\text{HMF}}=\frac{A_{266}-\varepsilon_{\text{LA}}^{266}C_{\text{LA}}}{\varepsilon_{\text{LA}}^{284}}. \end{array}$$

So, the content of LA and HMF in the hot acid hydrolysis solution of glucose can be calculated:

(9) $$\begin{array}{c} W_{\text{LA}}=C_{\text{LA}}*M_{\text{LA}}*K_{\text{LA}}*R,\\ W_{\text{HMF}}=C_{\text{HMF}}*M_{\text{HMF}}*K_{\text{HMF}}*R, \end{array}$$

where *W*_LA_, and *W*_HMF_ are the contents of LA and HMF in the hot acid hydrolysis solution, respectively, mg/L. *M*_LA_ and  *M*_HMF_ are the molecular weights of LA and HMF, respectively. *K*_LA_, and  *K*_HMF_ are the calibration coefficients of LA and HMF at wavelengths of 266 nm and 284 nm, respectively. *R* is the times of dilution.

### 3.5. Measurement Precision and Method Validation

The repeatability tests of the present method were conducted by adding some standard solutions of LA and HMF to a hot acid hydrolysis sample. The sample was measured by the present method, and the coefficients of recoveries of LA and HMF were calculated. The results are listed in Table 1. It can be seen that the repeatability of the method had a relative standard deviation of less than 4.47% for HMF and 2.25% for LA, and the recovery ranged from 88% to 116% for HMF and from 94% to 105% for LA, which can meet the requirement of rapid measurement for HMF and LA.

### 3.6. Application

According to the present method, seven samples were measured to determine the contents of LA and HMF, and the results were listed in Table 2. It can be seen that the maximum content of HMF would be achieved at 24 min with the hydrolyze reaction going along, the content gradually declining, and the content of LA begining to enhance and meeting the maximum at 29 min.

## 4. Conclusions

A very simple and rapid spectroscopic method to determine HMF and LA in hot acid hydrolysis solution of glucose had been developed. In this method, a sample was absorbed by charcoal, and direct UV absorption of the filtrate is then measured. The contents of HMF and LA can be calculated using a dual-wavelength (at 266 nm and 284 nm) spectroscopic technique. The present method is simple, rapid, and accurate and has the potential for online process monitoring.

## Figures and Tables

**Table 1:** Recovery test of the method.

Sample	Weight, *μ*mol					
	Added		Measured		Recovery, %	
	LA	HMF	LA	HMF	LA	HMF
1	34	148	32	130	94	88
2	50	123	49	144	98	116
3	78	165	84	173	108	104
4	97	200	102	188	105	94

**Table 2:** Contents of LA and HMF in the sample.

Reaction time, min	20	24	26	29	40	49	66
LA, g	0.32	1.39	1.40	2.00	1.80	1.87	1.49
HMF, g	0.27	1.06	0.38	0.46	0.17	0.11	0.05
